# Validation of HPLC method for the determination of chemical andradiochemical purity of a ^68^Ga-labelled EuK-Sub-kf-(3-iodo-y-) DOTAGA

**DOI:** 10.3906/kim-2003-19

**Published:** 2021-02-17

**Authors:** Ayşe UĞUR, Şükrü Gökhan ELÇİ, Doğangün YÜKSEL

**Affiliations:** 1 Department of Nuclear Medicine, Pamukkale University, Education and Research Hospital, Denizli Turkey; 2 Department of Biomedical Engineering, Faculty of Technology, Pamukkale University, Denizli Turkey; 3 Department of Nuclear Medicine, Medical Faculty, Pamukkale University, Denizli Turkey

**Keywords:** Imaging agents, radiolabeling, radiochemical purity, radio-HPLC, quality control

## Abstract

The prostate-specific membrane antigen (PSMA) represents an ideal biomarker for molecular imaging. Various PSMA-targeted radioligands are available for prostate cancer imaging. In this study, labeling of PSMA I&T with ^68^Ga, as well as validation of the radiochemical purity of the synthesis product by reverse phase radio high-performance liquid chromatography (HPLC) method are intended. Since the standard procedure for the quality control (QC) was not available, definition of chemical and radiochemical purity of ^68^Ga-PSMA I&T was carried out according to the Q2 (R1) ICH guideline. The standard QC tests were analyzed with Scintomics 8100 radio-HPLC system equipped with a radioactivity detector. The method was evaluated in terms of linearity, precision and accuracy, LOQ, robustness parameters, and specificity. To assess the radiochemical and chemical purity of ^68^Ga-PSMA I&T, the developed method was validated to apply safely to patients. An excellent linearity was found between 1μg/mL and 30 μg/mL, with a limit of detection and limit of quantitation of 0.286 μg/mL and 0.866 μg/mL, respectively for ^68^Ga-PSMA I&T. The recovery was 96.8 ± 3.8%. The quality control of the final product was performed many times with validated radio-HPLC method and was found to comply with ICH requirements, thus demonstrating the accuracy and robustness of the method for routine clinical practice.

## 1. Introduction

Prostate cancer is the most common cancer among men around the world. It is known to be the third most deadly form of cancer. However, correct diagnosis and staging of prostate cancer (PC) as in other types of cancer is very important for the effective treatment of patients. Among the techniques used in early diagnosis of cancer, sensitive imaging techniques have an important place. Positron emission tomography/computed tomography (PET/CT) with prostate-specific membrane antigen (PSMA) ligand has recently been offered to evaluate prostate cancer [1–3]. Radiolabeled PSMAs are the most important targets for imaging diagnostics and targeted radionuclide therapy of PC and its metastases due to their rapid and effective location in tumors [3,4]. In particular, gallium-68, as a radionuclide, is used for the labeling of PSMAs to obtain favorable imaging ligands for PET/CT-imaging. It shows higher tumor uptake and provides more acceptable background clarity. In this context, between ^68^Ga-PSMA 11, ^68^Ga-PSMA 617, and ^68^Ga-PSMA I&T, as the theranostic agents, ^68^Ga-PSMA I&T is more commonly used for PET/CT-imaging [5–7]. Szydlo et al. described the procedure for labeling PSMA ligands and quality control (QC) as a medicinal product in their study, and then compared the results of ^68^Ga-PSMA and 18F-fluorocholine [8]. PSMA radiopharmaceuticals labeled gallium-68 can be used to detect prostate cancer (PCa) cells due to excessive expression of PSMA. The ^68^Ga-HBED-PSMA ligand has been widely used and is considered the gold standard substance. McCarthy et al. examined whether PSMA I&T has comparative efficacy and they reported that PSMA I&T can replace PSMA-HBED as a diagnostic agent in prostate carcinoma [9]. According to Berliner et al., ^68^Ga-PSMA I&T PET/CT confirmed the potential for recurrent prostate cancer detection. ^68^Ga-PSMA I&T allowed morphological correlations to be detected by biochemical recurrence in approximately half of patients, even at low PSA levels below 0.5 ng/mL. The detection rate of ^68^Ga-PSMA I&T increased with increasing PSA levels to 100%. ^68^Ga-PSMA I&T detection rate was found to be comparable to that of ^68^Ga-HBED-CC [10]. ^68^Ga-PSMA I&T is preferred due to its high diagnostic accuracy for the detection of prostate cancer metastases, especially for lymph node metastases and at low PSA levels [11,12]. In addition, ^68^Ga-PSMA I&T shows good biodistribution and acceptable dosimetry without any toxicity in prostate cancer patients [13]. On the other hand, ^68^Ga-PSMA I&T, which is stable at room temperature and physiological pH, is easily chemically synthesized with a radiation safe procedure in a radioactive safe laboratory, using the commercially available PSMA I&T (EuK-Sub-kf-(3-iodo-y-), DOTAGA) [14]. Nowadays, ^68^Ga as a short-lived positron emitter (half-life 67.6 min) is produced using a 68Ge/^68^Ga generator [15,16]. The ^68^Ga solutions eluted from the earlier and state-of-the-art generators may be commonly contaminated with inorganic and/or organic residuals from the eluents used depending on the generator matrix. Therefore, the direct labeling procedures using the generator eluate may fail or result in low efficiency [17]. Ensuring that the final injectable radiopharmaceutical product fulfills regulatory requirements relating to contaminants, suitable production, and QC is crucial [18]. The standardization and validation of QC criteria are also a necessity to guarantee a high-quality radiopharmaceutical product like ^68^Ga-PSMA I&T, in particular in the routine clinical setting. However, according to our literature survey, no specific study about validating the QCs of ^68^Ga-PSMA I&T has been reported. In this regard, the QC of synthesized ^68^Ga-PSMA I&T in our laboratory is continuously carried out in terms of patient health. 

In the present study, we describe the validation of a reversed-phase radio-high-performance liquid chromatography (radio-HPLC) method used for the routine impurity control and quantification of synthesized ^68^Ga-PSMA I&T in our laboratory. The linearity, accuracy, precision, limit of detection (LOD), limit of quantification (LOQ), robustness, and specificity of the method were determined and evaluated in accordance with Q2 (R1) ICH guideline [19,20]. The validated method was applied to confirm the purity of PSMA I&T compared with the purchased reference material, as well as to test the QC criteria for routine use of ^68^Ga-PSMA I&T.

## 2. Experimental

### 2.1. Reagents

All reagents to be used for synthesis and QC were purchased from Merck in high purity pharmaceutical grade. Kit equipment for the synthesis of ^68^Ga peptides using cationic purification were obtained from ABX D-01454 Radeberg (Germany). The kit contains chemicals, hardware, and the cassette required for radiosynthesis of ^68^Ga peptides the Scintomics GRP synthesizer using cationic purification. The kit components are: Cassette, PS-H^+^ cartridge, 5M sodium chloride solution, ethanol, ethanol/water (1/1), phosphate buffered saline, 1.5M HEPES buffer solution, and water for injections. The cassette is a disposable cassette and therefore is made for single use. Reference PSMA I&T peptide was purchased from ABX D-01454 Radeberg (Germany) and stored at –20 °C. Dilutions of PSMA I&T were prepared with Farmako brand sterile water (1:1). Hydrochloric acid (0.6 M ultrapure HCl) and 1.5 M HEPES (2-[4-(2-hydroxyethyl)piperazin-1-yl]ethanesulfonic acid) buffer solution were obtained from ABX D-01454 Radeberg (Germany).

### 2.2. Instruments

**Figure 1:**
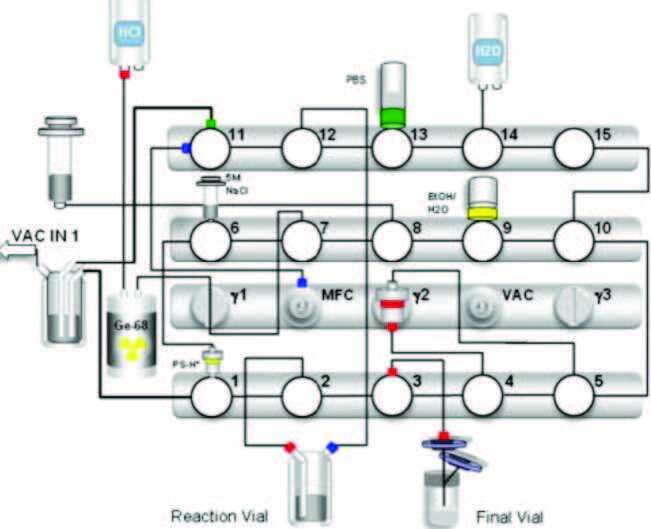

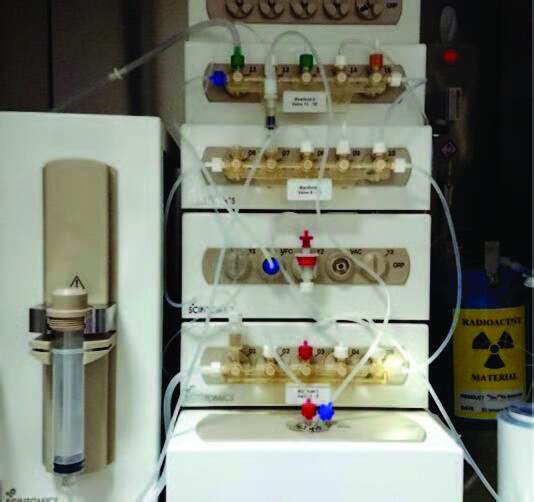
(a) Software interface module diagram of the automated Scintomics radiosynthesis system used to prepare 68Ga-PSMA I&T, (b) Original Scintomics radiosynthesis module equipped with cassette for the labelling of PSMA I&T peptide.

^68^GaCl_3_ was used for synthesis of ^68^Ga-PSMA I&T (Figure 1). ^68^Ga activity was measured in a Comecer VDC-606 dose calibrator. 

The radiochemical purity of ^68^Ga-PSMA I&T solution was controlled using Scintomics 8100 radio-HPLC system. The HPLC system was equipped with a UV and a gamma detector. The radio-HPLC system consists of Agilent 8100 quaternary pump (Germany) [Operating principle: parallel dual-plunger pump; low-pressure gradient, number of solvents: up to 4 solvents, gradient formation: 4-channel mixing valve, composition precision: <0.1%, composition accuracy: <0.5%], Welch Ultisil XB-C18 column (3.0 × 150 mm, 3 μm); SCI8120 UV/Visdetector (Germany) [Wavelength range: 190–900 nm, data collection rate: up to 50Hz, light source: Deuterium & Tungsten lamp, noise level: <±0.35.10^-5^ AU, 254 nm, dry cell, bandwidth: 5 nm, wavelength accuracy: ±1 nm, wavelength precision: ±0.1 nm, linearity: >99.5% for 2.5 AU (acetone, 254 nm), flow cell vol: 7 µL (analytical)], and radioactivity detector Berthold Technologies; DataApex Clarity program (Prague, Czech Republic).

### 2.3. Labelling of PSMA I&T with ^68^Ga in automated synthesis module

There is a specific synthesis cassette for the production of PSMA I&T and these cassettes are sterile and single-use. All components and the cassette are manufactured according to GMP Standards for APIs. In the studies, commercial 68Ge/^68^Ga generator modified with tin oxide (SnO2) was used in an iThemba Labs brand polyethylene column. A cation exchange cartridge (PS H^+^, not preconditioned) was used to remove trace metals in GaCl_3_ solution eluted from 68Ge/^68^Ga generator. GaCl_3_ eluted from the PS-H^+^ cartridge with 5.0 M NaCl was added to the reaction vial containing 25 µg peptide dissolved in HEPES buffer. The eluate was buffered with HEPES solution (pH 5) to prevent formation of ^68^Ga(OH)3 at higher pH values. The mixture was then heated for 15 min at 90 ºC for labeling of the peptide with ^68^Ga(III). The solution of ^68^Ga-PSMA I&T in the reaction vial was passed through the C18 ion exchange cartridge to remove the unbound free ^68^Ga ions. The retained ^68^Ga-PSMA I&T was eluted from the C18 ion exchange cartridge with 2.0 mL ethanol/water (1/1). The product was passed through the 0.22-μm filter syringe and collected in the final vial.

### 2.4. Quality control experiments

Th hydrophobic part of PSMA forms three diastereomers, RR, RS, and SS configurations at its amine nitrogens during chelating of gallium-68 with PSMA. The RR configuration is known as a thermodynamically favorable isomer. The other diastereomers have the same biological activity and radiochemical stability after the labeling procedure [21]. In addition to the requirements described above, a QC test is therefore required. The standard QC tests for ^68^Ga-PSMA I&T were carried out after each synthesis. The radiochemical purity of ^68^Ga-PSMA I&T solution was identified with Scintomics 8100 radio-HPLC system equipped with a radioactivity detector, under working conditions maintained with a flow rate of 0.6 mL/min and column temperature at room temperature. An isocratic separation was performed using a mobile phase including 30% acetonitrile and 0.1% trifluoroacetic acid (TFA) in water. For the detection of chemical impurities, the samples were also monitored with a UV detector at 220 nm and radio-detector. The injection volume was 20 μL and detection time determined as 15 min.

## 3. Results and discussion

### 3.1. Labelling of PSMA I&T with ^68^Ga 

All synthesis studies were conducted in a radiopharmaceutical laboratory at Department of Nuclear Medicine, in Pamukkale University Hospital. The synthesis of ^68^Ga-PSMA I&T was performed a total of 150 times between March 2018 and January 2019 in our laboratory. The reagent and hardware kit manufactured by ABX for Scintomics is produced after bioburden testing. All reagents used are sterile and the synthesis process is carried out under GMP conditions. Therefore, no additional QC is required for method validation. 

The activity of the ^68^Ga gallium labeled PSMA peptide depends on the activity eluted from the 68Ge/^68^Ga generator. For the 30 mCi of eluate the Scintomics GRP automated synthesis module can produce around 18–20 mCi of product. At the end of synthesis, the total volume of the final product is 16 mL. The labeling efficiency was found to be >99%. The automated synthesis was performed within 32 min. Postsynthesis of the product after 45 min (after QC) remained at a total activity of 68% that is convenient for clinical application. The pH of the final product was determined to be in between 6 and 7.

As the synthesis yield decreased, the dose and number of patients were adjusted. From the synthesis of the 68Ge/^68^Ga generator in the first months, sufficient activity was obtained for three patient doses (185 MBq to 259 MBq per dose).

### 3.2. Physical and chemical properties of ^68^Gallium and PSMA I&T

**Figure 2:**
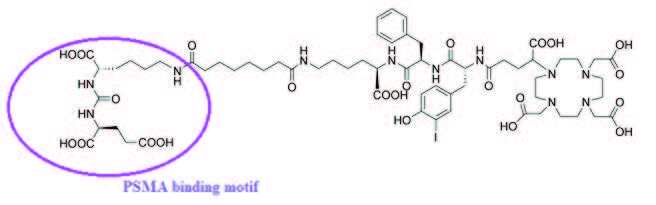
EuK-Sub-kf-(3-iodo-y-)DOTAGA(PSMA I&T) chemical structure [3].

^68^Ga(III) as a positron emitter is readily obtained from modern generators and has a short half-life of 68 min [22]. It is used for labeling ligands having five or six coordination sites and the labeled complexes are stable at the physiological pH. Like metallic elements, transition metals also require chelating agents. After labeling a molecule with a metal or transition metal chelator, a molecular structure appears whose final chemical structure is affected by the chelate. Therefore, large molecules such as peptides, proteins, or antibodies are preferred over radiolabeling with metal or transition metal. For radiopharmaceutical preparation with metal or transition metal, the appropriate chelating agent must be present for each metal or transition metal. Gallium is in the IIIA group of the periodic table. It is in the form of three ions and a chelation such as 1,4,7-triazacyclonona-1,4,7-triacitic acid (NOTA), 1,4,7,10-tetraazacyclododecane-1,4,7,10-tetraacetic acid (DOTA) is required [23,24]. These two chelating agents are widely used in routine PET imaging with ^68^Ga. Prostate-specific membrane antigen (PSMA) is the biological target for therapeutics and diagnostics. It is a type II transmembrane protein with a short intracellular area (amino acids 1–18), a transmembrane area (amino acids 19–43), and a large extracellular area (amino acids 44–750) [25–28]. Also, PSMA ligands with DOTA-derived chelators have been developed, which can be labeled with diagnostic compounds. Recently, EuK-Sub-kf-(3-iodo-y-) DOTAGA (PSMA I&T) (Figure 2) from these diagnostic nuclides is of interest.

### 3.3. Discussion of quality control

Although the ^68^Ga-PSMAI&T synthesized for the patient has a thermodynamically preferred diastereomer, the other diastereomer is also known to be present in the formulation. Thus, there is a need for a QC test to assess the diagnostic efficiency and purity of the final product. The QC was evaluated with the analysis carried out by high-performance liquid chromatography (HPLC). 

### 3.4. Validation of the HPLC method 

The analytic method validation for the defined chemical and radiochemical purity of ^68^Ga-PSMA I&T was carried out according to the Q2 (R1) ICH guideline. The most important performance parameters for the validation were evaluated as linearity, precision, accuracy, limit of quantification (LOQ), robustness, and specificity [29].

**Figure 3:**
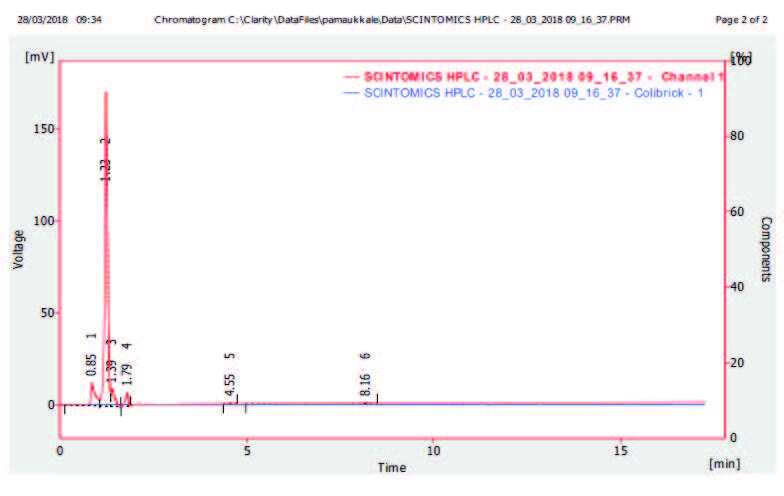

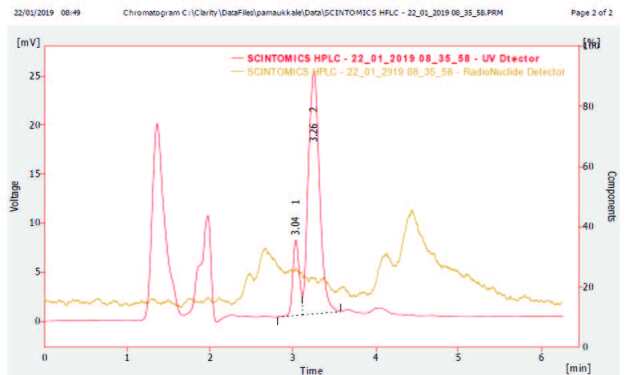

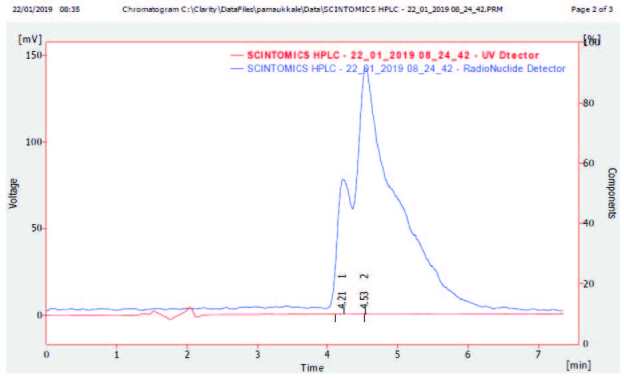
HPLC chromatograms of blank chromotogram (a) PSMA I&T (b) and ^68^Ga-PSMA I&T (c) obtained with UV and radionuclide detectors, respectively.

Under the chromatographic conditions described in the experimental section the blank chromatogram was shown in Figure 3a, PSMA I&T chromatogram (Figure 3b) and ^68^Ga-labeled PSMA I&T chromatogram (Figure 3c) were used for analytical HPLC method validation. When comparing the chromatograms of PSMA I&T and ^68^Ga-PSMA I&T obtained with the UV and radionuclide detector, respectively, it is understood that the peak intensities and retention times of the first and second isomers in both chromatograms are relatively similar. The average retention time of ^68^Ga-PSMA I&T was 4.53 min using a radionuclide detector (Figure 3c). 

We believe that the broad peak range of ^68^Ga-PSMA I&T in the radiochromotogram is the continuation of radioactive radiation generated by the radioactivity passing through the column, and the presence of this radiation by the radionuclide detector.

It turned out that minor radiochemical impurities are formed (altogether approximately 1%), which could not be separated by HPLC. Significant chemical impurities were not observed in any of the batches produced by the described method.

#### 3.4.1. Linearity

**Figure 4:**
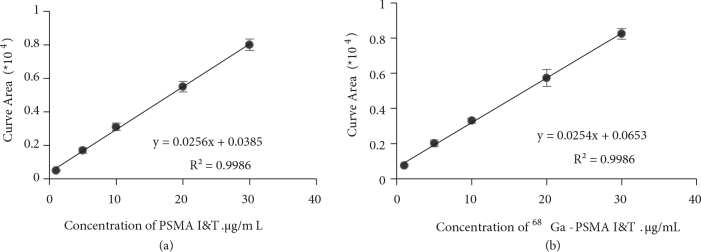
Calibration curves for (a) PSMA I&T and (b) ^68^Ga-PSMA I&T using the average values of peak areas (n = 5).

Calibration standard solutions of ^68^Ga-PSMA I&T and PSMA I&T were prepared in the range of 1 and 30 μg/mL and then they were injected into the HPLC systems under the above specified chromatographic conditions (injection volume was 20 μlL). The calibration curves were plotted for the peak areas versus ^68^Ga-PSMA I&T and PSMA I&T concentrations and their regression equations were computed (Figure 4). The regression equations for PSMA I&T and ^68^Ga-PSMA I&T were found to be y = 0.0256x + 0.0385 (R_2_ = 0.9986) and y = 0.0254x + 0.0653 (R_2_ = 0.9986), respectively. The good correlations for both species were found to be ≥0.99% and met acceptance criteria. 

#### 3.4.2. Precision and accuracy 

While preparing three different concentrations of ^68^Ga-PSMA I&T ranging from 1.0 to 30 µg/mL, an aqueous solution of peptides was prepared. The calculated amount of peptide was taken with a micropipette, and labeling was done with ^68^Ga. Three different concentrations of ^68^Ga-PSMA I&T were chosen and injected into the HPLC system. Intraday precision and accuracy using the regression equation were calculated by five replicates for each concentration (Table 1). According to the ICH validation guideline, the low value for relative standard deviation (RSD%, <2%) reflects the high precision of the method. 

**Table 1:** Intraday precision and accuracy for determination of ^68^Ga-PSMA I&T using the aimed RP-HPLC method (n = 5).

Concentrations of ^68^Ga-PSMA I&T, µg/mL			Standarddeviation	RSD	Recovery, %
Actual(Prepared)	Foundvalues	Calculatedaverage			
1	0.93				
	0.85				
	0.89	0.932	0.066	0.071	93.2 ± 7.1
	1.02				
	0.97				
					
	9.63				
	9.24				
10	9.94	9.648	0.842	0.087	96.5 ± 8.7
	8.58				
	10.85				
					
	30.89				
	30.57				
30	28.53	30.258	0.975	0.032	100.8 ± 3.2
	30.73				
	30.57				

The accuracy was expressed by the percentage of recovery (R%) calculated as the ratio of found concentration to actual concentration. The quantitative recovery values that are higher than 95% are the accepted criterion for ​​expressing high accuracy. In these workings the recoveries were calculated in range of 93.2 ± 7.1 and 100.8 ± 3.2% (n = 5) with the average recovery of 96.8 ± 3.8 (Table 1). This result shows that the average recovery is higher than the 95% required to show high accuracy of the method. For 30-μg peptide, the relative standard deviation (RSD) value of average recovery (3.2%) shows that the precision is moderately high. 

#### 3.4.3. Limit of detection (LOD) and limit of quantitation (LOQ)

The LOD and LOQ values are determined by the analysis of samples with known concentrations of analytes at the minimum level (here, 1 µg/mL) in which the analyte can be reliably detected. Standard solutions were prepared at concentrations less than 1 μg/mL, the lowest value of the calibration curve. Chromatograms of these solutions were taken and the slope of the calibration curve and calculated using 3.3 ϭ/S and 10 ϭ/S equations, respectively, where ϭ is the standard deviation of the response and S is the slope of the calibration curve. LOD and LOQ values were calculated as 0.586 and 1 µg/mL for PSMA I&T, and 0.866 and 1 µg/mL for ^68^Ga-PSMA I&T, respectively.

#### 3.4.4. Robustness

The variation parameters used to evaluate the robustness of the developed method depends on the type of procedure under investigation. In this investigation, to study the robustness of the method, some changes were made in the flow rate and the composition of mobile phase. To study the robustness of the method, the effect of flow rate and the composition of mobile phase on ^68^Ga-PSMA I&T determination by the proposed RP-HPLC were investigated (Table 2). Flow rate was changed by ±0.1 unit. The mobile phase components at three different concentrations were injected in triplicates. Depending on relative standard deviation values ​​reflecting high precision in Table 2 (RSD% < 2, except two of them), the relative standard deviation value (RSD% = 0.4%) found for 30-µg peptide determination using a mobile phase including 30% acetonitrile and 0.10% trifluoroacetic acid ) at 0.6 mL/min flow rate confirms the robustness of the method.

**Table 2:** Effect of mobile phase composition and flow rate on ^68^Ga-PSMA I&T determination by the proposed RP-HPLC (n = 3).

Parameters	Variations	Area (mV.s)(*10,000)	RSD,%
Flow rate,a mL/min	0.5	0.994	1.4
		1.013	
		1.021	
	0.6	0.842	0.4
		0.846	
		0.848	
	0.7	0.559	2.2
		0.584	
		0.569	
Trifluoroacetic acid conc.,%b	0.09	0.674	1.4
		0.693	
		0.683	
	0.10	0.842	0.4
		0.846	
		0.848	
	0.11	0.733	0.6
		0.732	
		0.725	
Acetonitrile conc.,%c	27	0.810	2.0
		0.790	
		0.822	
	30	0.842	0.4
		0.846	
		0.848	
	33	0.655	3.5
		0.665	
		0.622	

aAcetonitrile 30% and Trifluoroacetic acid 0.10% into water, bAcetonitrile conc. was held constant as 30%. cTrifluoroacetic acid conc. was held constant as 0.10%, b,cThe flow rates were 0.6 mL/min.

#### 3.4.5. Specificity

Specificity determination is performed by analyzing the mixture containing crucial components that might be present in the final product ^68^Ga-PSMA I&T solution. The method is capable of discriminating the various components present at the limited concentration for the considered standards. Analyses were performed using a series of solutions containing ^68^Ga-PSMAI&T at different concentrations (Table 1). The concentration changes had no effect on the average recovery that was 96.8 ± 3.8% (RSD%: 3.9%, n=3) calculated from Table 1, therefore the method was found to have acceptable specificity for assay of ^68^Ga-PSMA I&T with high accuracy.

## 4. Conclusions

Here, we developed and validated a simple and rapid isocratic radio-HPLC method with high precision for quality control testing of synthesized ^68^Ga-PSMA I&T radiopharmaceuticals for its use in routine PET imaging in prostate cancer patients. The absence of specific literature about the quality controls of ^68^Ga-PSMAI&T has led us to use isocratic radio-HPLC method for validation of the produced radiopharmaceutical to implement it in the clinical setting.

The bioburden and endotoxin levels of the 0.05 M hydrochloric acid are controlled and specified on the certificate of analysis. We consider the verification of this certificate to be sufficient; therefore, we did not perform a routine assay for bioburden or bacterial endotoxins on the ^68^Ga eluate.

Definition of chemical and radiochemical purity of ^68^Ga-PSMA I&T was carried out according to the Q2 (R1) ICH guideline. The method was validated in terms of linearity, precision and accuracy (recovery), LOD and LOQ, robustness, and specificity parameters. In addition, samples can be analyzed quickly due to the short analysis time (less than 10 min) required. As a result, it was concluded that the proposed method was applicable and reliable in determining the radiochemical purity of ^68^Ga-PSMA I&T from the validation results obtained in this study.
